# Determinants of Successful Exclusive Breastfeeding for Saudi Mothers: Social Acceptance Is a Unique Predictor

**DOI:** 10.3390/ijerph18105172

**Published:** 2021-05-13

**Authors:** Nada A Alyousefi

**Affiliations:** 1 Department of Family and Community Medicine, College of Medicine, King Saud University, Riyadh 12372, Saudi Arabia; nalyousefi@ksu.edu.sa; Tel.: +966-114670836; 2 King Saud University Medical City, King Saud University, Riyadh 12372, Saudi Arabia

**Keywords:** exclusive breastfeeding, awareness, professional lactation support, infant nutrition, social acceptance

## Abstract

Recent guidelines motivate health care professionals to promote exclusive breastfeeding (EBF). The reported rate of EBF is low in Saudi Arabia. This study aimed to explore the determinants of successful exclusive breastfeeding for Saudi mothers. A cross-sectional, survey-based study was conducted in family medicine clinics. The dependent variable was the actual practice of EBF. Independent variables were the mothers’ demographic information, comfortableness with breastfeeding in public, knowledge and attitudes about breastfeeding, previous experience of successful breastfeeding, and a previous feeding plan. Statistical analysis was carried out using bivariate analysis and multinomial logistic regression. Out of the 322 respondents, only 28% practiced exclusive breastfeeding for the first six months. Perceived insufficient milk (*p* = 0.011) was associated with a lower EBF rate. Mothers’ degrees of comfort with breastfeeding in front of their relatives (*p* = 0.024) and in front of friends (*p* = 0.028) were significantly associated with their infants’ actual feeding practices for the first six months of their infants’ lives. Mothers reported that the absence of a suitable place for breastfeeding caused them to stop breastfeeding (*p* = 0.043) and was associated with their infant’s actual feeding practices for the first six months of their infant’s lives. An antenatal breastfeeding intention was considered a significant predictor of EBF; OR: 7.31 (95% CI: 2.24—23.84). Mothers who do not stop breastfeeding when they get sick have a 5.054 times higher chance of continuing EBF (95% CI: 1.037—24.627) than the formula-only feeding group. Thus, social acceptance is a unique predictor for their success in exclusive breastfeeding. Mothers have good intentions and a desire to breastfeed. Therefore, they must be guided through their pregnancy and postpartum period to overcome breastfeeding issues.

## 1. Introduction

Among the essential components of maternal experiences is breastfeeding. It has been recommended by the World Health Organization (WHO) that breastfeeding should be continued for at least two years, with the beginning of weaning and addition of foods after six months of the initial period of exclusive breastfeeding [1].

Recent guidelines and newborn breastfeeding-friendly initiatives [2,3] have been implemented in the community to help health care professionals to promote the awareness and practice of breastfeeding among mothers [4]. Breastfeeding has gained variable attention from health care policymakers, practitioners, and mothers despite all of the proven benefits and promotional efforts.

The breastfeeding rate has declined in different areas, including urban and rural [5,6,7]. In developing countries, less than 40 percent of infants under six months of age were exclusively breastfed [8]. In Egypt, the rate of exclusive breastfeeding was reported to be 42.5 percent among infants aged four months. A study in Dhaka reported that 53 percent of exclusive breastfeeding was observed at one month of age; it declined to 5 percent at six months of age [9]. In Iran, 27.7 percent and 56.8 percent at six and four months of infants were reported, respectively [10]. This is possibly affected by breastfeeding knowledge and factors such as occupation, culture, education, and the mother’s socioeconomic status [11].

The literature has shown that Saudi mothers are willing to breastfeed, as a relatively high breastfeeding prevalence at birth has been observed [12]. Breastfeeding continuation is still an issue. The rate of exclusive breastfeeding reported in different cities in Saudi Arabia is still low. Studies that used the WHO definition of exclusive breastfeeding in Saudi Arabia reported that the exclusive breastfeeding rate at six months of age ranged from 1.7% [13] to 37% [14].

The factors contributing to the continuation of breastfeeding, which varies from country to country [5,15,16,17,18], included mothers with no plans to breastfeed longer than six months, not attending any training sessions, and an infant receiving formula milk in the hospital. As described by some studies [15,18,19,20,21], reasons for the discontinuation of breastfeeding might include maternal age, educational background, socioeconomic status, postpartum depression, maternal confidence, maternal obesity, and being overweight [22], whereas factors associated with a higher breastfeeding rate and longer duration include increased maternal age, low educational levels, rural residence, low income, multiparity, and avoiding contraceptives [23].

There has been a shift in women’s roles in Saudi society from a solely motherhood role to a multirole with higher education, employment status, and socialization [24,25,26,27,28]. A published study found that full-time working mothers’ support timing is essential, including acute support, such as establishing a successful latch needed during the first two weeks after delivery and the necessary long-term support to overcome breastfeeding issues [29]. A study conducted in Saudi Arabia showed that an early return to work, deficient breastfeeding work support, and lack of time were the major barriers to exclusive breastfeeding in primary healthcare working mothers in Al-Ahsa [20].

A careful understanding of these impacts on exclusive breastfeeding is vital in order to plan and implement effective interventions in this regard. This study aimed to explore the determinants of Saudi mothers for successful exclusive breastfeeding.

## 2. Materials and Methods

A cross-sectional study was conducted in family medicine clinics at King Saud University Medical City (KSUMC) in Riyadh in 2019. KSUMC is among the governmental university-based hospitals in Riyadh. Eligible mothers for the study were Saudi, Arabic speakers with infants between 6 months and 24 months of age who presented to family medicine clinics of KSUMC for vaccination.

Considering the total registered infants of 6–24 months old at the clinics with the published prevalence of exclusive breastfeeding in Saudi Arabia of 37% [14], the sample size (*n*) was calculated according to the following formula: *n* = z2 * *p* * (1−*p*)/e2, where: z = 1.96 for a confidence level (α) of 95%, *p* = proportion (expressed as a decimal), e = margin of error. z = 1.96, *p* = 0.37, e = 0.05 *n* = 1.962 * 0.37 * (1−0.37)/0.052 *n* = 0.8955/0.0025 = 358.191 *n* ≈ 359. The sample size was calculated to be 359. The simple random sampling technique was used.

The dependent variable was exclusive breastfeeding (EBF). Its definition was breastfeeding only, with no other food, liquid, or water, except drops or syrups consisting of vitamins, mineral supplements, or medicine. The used definition of mixed feeding is as follows: the infant was fed with breast milk, infant formula, and other types of food. The used definition of exclusive infant formula feeding is as follows: the infant was fed with infant formula but without any breast milk. Those definitions were used by other authors in their publications [30,31,32]. Independent variables of the study, including the demographic information, such as the mother’s age, mother’s employment status, educational level, income, and presence of any chronic illnesses, were included.

The author developed the questionnaire based on a thorough literature search [21]. The questionnaire explored whether the mothers would be comfortable breastfeeding in front of close women friends, close male relatives, female friends, or the public using “Agree”, “Disagree”, or “Not sure”. The questionnaire explored whether a mother implemented formula feed, mixed feed, or breastfeeding. Whether she agreed or disagreed with a series of evidence-based statements about breastfeeding and child health was assessed using “Agree”, “Disagree”, or “Not sure”. Questions regarding the previous experience of successful breastfeeding, previous plans, and decision-making timing for feeding type were included. The questionnaire was pretested on a similar sample of 25 mothers, which was not included in the actual survey.

Ethical approval was obtained from the institutional review board, College of Medicine, King Saud University. The questionnaire contained informed consent indicating the purpose of the study and the participant’s right to withdraw at any time without any obligation towards the study team.

Statistical analysis was carried out using the SPSS (IBM Corp. Released 2012. IBM SPSS Statistics for Windows, Version 21.0. Armonk, NY: IBM Corp.). Bivariate analysis was carried out to identify factors associated with exclusive breastfeeding. The multicollinearity among the independent variables was checked by creating a correlation matrix. The absolute value of Pearson correlation showed no evidence of multicollinearity. Multinomial logistic regression was carried out to quantify the association between significant variables in the bivariate analysis and mothers’ actual practice of exclusive breastfeeding, mixed feeding, or exclusive formula feeding. The model fitness was tested using Cox and Snell and Negelkerker R2. The associations were reported by the odds ratio. The statistical tests with *p*-values of less than 0.05 were considered statistically significant.

## 3. Results

The survey had an overall response rate of 89.7% of 359 recruited mothers. Table 1 showed participants’ sociodemographic characteristics and their associations with their actual breastfeeding practice for the first six months of their infants’ lives. Most of the studied mothers were between 25 and 35 years old. Most of them were college graduates (49.4%) and healthy, without known chronic diseases (85.8%). About 59% were housewives, and most of them (54%) had a family income of SAR 5–10 thousand per month, which is equal to USD 1333–2666. Most of them delivered their last child through normal vaginal delivery (75%).

More than half of the participants (53.7%) practiced mixed feeding. The rate of exclusive breastfeeding for the first six months was 28%. The proportion of mothers who used formula feeding was 18.3%. Only 36.2% had previous experience of breastfeeding. Most of them planned to do a mixed type of feeding (71.4%). Most of them decided to do so before getting pregnant (44.4%).

Age, the educational level of the mother, occupation status, family income, the mode of delivery, and chronic maternal illness were not significant predictors of exclusive breastfeeding practice. The study found that the presence of previous breastfeeding experience and the prior intention or plan of breastfeeding were possible predictors of infant feeding practice.

Table 2 shows Saudi mothers’ knowledge about the health effects of breastfeeding, attitudes, and actual practice of feeding infants for the first six months of their lives. About two-thirds of the participants (68.9%) felt that they could overcome breastfeeding problems, and 73.3% knew the WHO definition of exclusive breastfeeding for the first six months of infants’ lives. Both items were not significant in determining their actual practice.

About seventy-six percent (76.1%) of participants knew that breastfeeding helps them to get rid of additional weight that gained during the pregnancy period, helps the uterus return to its normal position faster (89.7%), contributes to preventing breast and ovarian cancer (85.1%), assists in contraception (58.4%), helps to enhance the relationship and increase familiarity with the child (93.1%), contributes to improving body image (56.5%), and helps to improve general health (78%). In this knowledge part, there was not any significant correlation with mothers’ actual practice.

In regard to items exploring mothers’ attitudes and knowledge about breastfeeding, the study found that about half of the mothers might stop exclusive breastfeeding if the milk is insufficient (51.9%, *p*-value = 0.011) and if they get sick (39.1%, *p*-value = 0.000).

Maternal agreement with other statements, as shown in Table 2, did not predict their success in exclusive breastfeeding, such as problems with the nipple or breast that may force them to stop breastfeeding (39.5%); the possibility of breastfeeding distorting breast shape (21.1%); breastfeeding being more clean, easy, and available (91.4%); breastfeeding being more economical and less expensive than formula milk (83.2%); and breastfeeding infants being less prone to getting diarrhea (85.4%), ear infections (86.9%), chest infections (78%), constipation (85.1%), colds (72%), food allergies (72.1%), and asthma (65.9%); and being smarter than infants who receive formula feed (66.5%), in addition to being quieter (57.1%), healthier (86%), and less obese (69.3%).

Table 3 shows the associations among the three items concerning mothers’ degrees of comfort when breastfeeding in social situations, i.e., in front of relatives (46.2%), (*p*-value = 0.024); close woman friends (45.3%), (*p*-value = 0.028); or in public (16.4%), (*p*-value = 0.132), with their infant’s actual feeding practices for the first six months of their infant’s lives. It also shows the associations between maternal comfort when breastfeeding in social situations and success in exclusive breastfeeding. Whereas 69.2% agreed that our society promotes and supports breastfeeding practice, this was not a predictor of their actual practice. On the other hand, 49.7% of mothers agreed that the absence of a suitable place for breastfeeding caused them to stop breastfeeding. This predictor was found to be significant (*p*-value = 0.043).

Table 4 shows further evaluation with multinominal logistic regression analysis for the possible predictors of successful exclusive breastfeeding experience. A mother with an antenatal plan of exclusive breastfeeding compared to an antenatal plan of mixed feeding will be more likely to practice exclusive breastfeeding at the age of 6 months, i.e., OR: 7.31 (95% CI: 2.24–23.84), than mothers who practice exclusive formula feeding, whereas mothers with an antenatal plan of exclusive formula feeding compared to an antenatal plan of mixed feeding will be less likely to practice exclusive breastfeeding at six months, i.e., OR: 0.041 (95% CI: 0.005–0.367), than mothers who practice exclusive formula feeding. Mothers who do not stop breastfeeding when they get sick will have a 5.054 times higher chance of continuing exclusive breastfeeding (95% CI: 1.037–24.627) compared to the mothers in the formula-only feeding group. Mothers with an antenatal plan of exclusive formula feeding compared to the antenatal plan of mixed feeding will be less likely to practice mixed feeding, i.e., OR: 0.013 (95% CI: 0.002–0.112), than mothers who practice exclusive formula feeding.

## 4. Discussion

Most participants practiced mixed feeding, i.e., partial breastfeeding combined with bottle feeding. Mixed feeding is prevalent among Saudi mothers compared to exclusive breastfeeding [33].

In this study, the rate of exclusive breastfeeding for the first six months was 28%. The breastfeeding rate in Al-hassa (a city in the eastern region of Saudi Arabia) at birth was reported at 76.1% and declined to 12.2% at the age of six months [16]. In a study conducted in Riyadh, only 37.5% of the mothers practiced exclusive breastfeeding for six months [34]. Exclusive breastfeeding rates were 33.1% and 27.3%, respectively, in Dammam and Al-Kharj [23,35].

This is almost similar to the reported exclusive breastfeeding rates in Sub-Saharan Africa and East Asia/Pacific, which demonstrated an upward trend in the last decade from 24% to 32% and from 27% to 32%, respectively [36]. For example, it was 33.7% in the Philippines, 38.9% in Indonesia, and the highest in Cambodia, i.e., 60.1% [19]. Exclusive breastfeeding in the first six months of birth in the United States among infants born in 2017 was 25.6% [37].

The rate of breastfeeding regarding ethnic differences has been reported to be high among Asian women [38]. There is evidence that the rate of breastfeeding varies according to socioeconomic and demographic factors. As reported in some studies, rural, less-educated, low-income, multiparous mothers were more likely to exclusively breastfeed their infants [16]. Although the study did not find a significant association, generally, exclusive breastfeeding was influenced by socio-demographics, especially maternal educational and employment status [16]. Higher education was associated with shorter breastfeeding in a Saudi study [16]; however, in other industrialized nations, mothers with higher education levels have improved breastfeeding outcomes compared to their less-educated counterparts [39]. Most participants of our sample were urban and highly educated, making it challenging to conclude.

In line with the WHO recommendations, 73.3% of the study participants were aware of the definition of exclusive breastfeeding (EBF). This result supports the implementation efforts carried out by Saudi health institutes of the ten steps of the Baby-Friendly Hospital Initiative [3], which encourages improving the knowledge, attitudes, and practice of exclusive breastfeeding. The majority of mothers were aware of the advantages of EBF. Out of the 322 respondents, 294 (91.4%) believed that breastmilk was cleaner and safer. Only 21.1% of our participants thought that breastfeeding might distort their breast shape. This result contrasts with a study conducted in 2007 [40], where 70.8% of their respondents were reportedly afraid of breastfeeding because they feared losing their attractive figures. Furthermore, 83.2% of our participants had positive attitudes towards the cost of breastfeeding.

In this study, previous successful breastfeeding experiences for an earlier child were significant for successful exclusive breastfeeding. In accordance with the study findings, a Saudi study showed that Saudi mothers are willing to breastfeed [12]. A study conducted in Australia indicated that the factors related to breastfeeding continuation comprise a very firm desire to breastfeed [41]. The same finding was evident in this study. Mothers with previous plans and intentions for exclusive breastfeeding were more likely to succeed in exclusive breastfeeding.

Another significant reason to fail in breastfeeding continuation is the mother’s belief that she has insufficient breast milk. This was reported by about half of the sample in some studies [33,42]. In a Saudi study, insufficient breast milk was the most common reason for stopping breastfeeding (25.9%) [34]. The mechanism of perceived insufficient breast milk could be explained by less breast stimulation and less milk secretion due to reduced sucking of the breast when introducing bottle feeding [43,44].

Although physiological deficiency can occur, it is probably not the leading cause of perceived milk insufficiency [35]; some mothers thinking that they have insufficient breast milk does not lead to it being the most common reason to stop breastfeeding and introduce formula feeding [45]. Therefore, insufficient breast milk as a reason to practice mixed feeding may be more perceived than real.

Another reason to stop breastfeeding is the sickness of the mother. Some mothers think that taking medicines will interfere with breastfeeding. This reason was given less frequently but varied considerably in mothers’ proportion across the published studies [23] and was considered significant in this study. In the Infant Feeding Practices Study II (IFPS, II), which was a longitudinal study of mothers of infants that was conducted from 2005 to 2007 by the U.S. Food and Drug Administration and the Centers for Disease Control and Prevention, mothers who got sick or had to take medicine were 2.2 times (OR: 1.51–3.26) more likely to stop breastfeeding [46,47].

Social acceptance can act as a significant barrier to breastfeeding, including embarrassment at breastfeeding in front of others, even of the same gender [48]; fears of the evil eye [45] (superstitious fears of envy of the lactating mother, including refusal of breastfeeding, cessation of milk flow, or disease for the nursing infant); or lack of special facilities, such as lactation rooms in public places [16]. Although for decades before civilization, breastfeeding was the only way to feed infants, this is not only limited to the Saudi population. In the U.S., a survey found that women who felt comfortable breastfeeding in public intended to exclusively breastfeed for longer than those who felt uncomfortable [21].

Furthermore, in Saudi society, breastfeeding in public is considered difficult for some mothers, especially with modesty and the absence of a suitable place for breastfeeding in public. Additionally, the lack of family support can overshadow some advantages of breastfeeding. There has been a considerable change in the role of women in Saudi society. Women’s traditional, solely motherhood role has shifted to a multirole with more socialization outside the home, with higher education and careers that need further evaluation and interventions to facilitate caring especially for employed Saudi mothers.

An additional constraint for employed women includes the relatively short maternity leave (about 45–70 days), which may force Saudi women to hire babysitters, who are often the predominant feeders of the children [49]. For breastfeeding interventions to be successful, they should explore the community perceptions and social norms that shape women’s decisions to continue breastfeeding [49]. Improving the availability of certified lactation consultants and trained breastfeeding educators in Saudi Arabia is essential for successful interventions.

Among the study’s limitations is that mothers who breastfed 24 months before the survey were included with those who breastfed for only six months. While this is common in the design of breastfeeding studies, there is a possibility of recall bias. This was minimized by excluding mothers who had a child who was older than 24 months. Future research in the form of cohort studies with appropriate follow-up data to examine the actual feeding practice to avoid mothers’ recall bias and explore all possible factors and confounders can provide more accurate results. The study results cannot be generalized to Saudi society. Nevertheless, they elucidate the important factors related to breastfeeding, contributing to improving childhood and motherhood outcomes. Interventional research could be the next step in implementing the recommendations provided by this study.

## 5. Conclusions

The majority of the participants were aware of exclusive breastfeeding and believed that the practice was desirable and low cost. They knew that breastmilk alone is sufficient for the baby for the first six months, yet only less than two-thirds of them practiced exclusive breastfeeding. The reported rate of breastfeeding initiation and exclusivity are far lower compared to the current WHO recommendations.

Maternal comfort when breastfeeding in some social settings was directly related to the intention to exclusively breastfeed. Antenatal interventions that address these issues may increase exclusive breastfeeding intention and duration. Irrespective of maternal educational status, many misconceptions are prevalent regarding breastfeeding practices. The return to a breastfeeding culture should be encouraged with community involvement.

There is a declining trend observed in breastfeeding practices, regardless of the significant advances of Saudi Arabia in health services. Professional lactation support can help mothers initiate and continue breastfeeding. There is a need to promote, support, and maintain exclusive breastfeeding practices as part of public health strategies to decrease infants’ and mothers’ health risks in Saudi Arabia.

The Saudi Ministry of Health (MOH) will implement several health initiatives related to the National Transformation Program (NTP) 2020 and the Saudi Vision 2030 [50]. Among the initiatives concerns maternal and child health is the Women’s Health awareness platform, which addresses important topics, including breastfeeding. MOH has also given attention to safe child feeding and has established the Breast Milk Substitutes Marketing Law and its implementing regulations [51].

Breastfeeding rooms started to be placed in some workplaces and public spaces in Saudi Arabia. Training and increasing the numbers of certified lactation consultants with a more defined role in Saudi health institutes can be a key factor in protecting breastfeeding in the Saudi community. The recent announcement of the establishment of the Saudi breastfeeding association is an example of ongoing efforts that are on the right track.

## Figures and Tables

**Table 1:** Sociodemographic, health-related characteristics, and practices of the sample of surveyed mothers in relation to their feeding patterns for their infants for the first six months of their infants’ lives in Riyadh, Saudi Arabia.

*p*-Value		Number (%)		
	Formula Feeding Only	Mixed Feeding	Exclusive Breastfeeding (EBF)	Variables
	59 (18.3)	173 (53.7)	90 (28)	Overall 322 (100)
0.703	Mother’s Age			
	1 (0.3)	2 (0.6)	1 (0.3)	<20
	13 (4)	46 (14.3)	21 (6.5)	20–25
	22 (68)	53 (16.5)	28 (8.7)	26–30
	14 (4.3)	44 (13.7)	26 (8.1)	31–35
	9 (2.8)	28 (8.7)	14 (4.3)	>35
0.076	Mother’s Education Level			
	0 (0)	3 (0.9)	5 (1.6)	Uneducated
	4 (1.2)	9 (2.8)	3 (0.9)	Primary School
	5 (1.6)	5 (1.6)	5 (1.6)	Intermediate School
	14 (4.3)	55 (17.1)	34 (10.6)	Secondary School
	31 (9.6)	91 (28.3)	37 (11.5)	College Graduate
	5 (1.6)	10 (3.2)	6 (1.9)	Post-graduate
0.287	Mother’s Chronic Illness			
	6 (1.9)	25 (7.8)	15 (4.7)	Yes
	53 (16.5)	148 (46)	75 (23.3)	No
0.132	Mother’s Occupation			
	5 (1.6)	17 (5.3)	5 (1.6)	Student
	22 (6.8)	57 (17.7)	26 (8.1)	Employee
	32 (9.9)	99 (30.7)	59 (18.3)	Housewife
0.949	Family Monthly Income			
	9 (2.8)	25 (7.8)	15 (4.7)	<5000 Saudi Riyals
	33 (10.2)	94 (29.2)	47 (14.6)	5000–10,000 Saudi Riyals
	11 (3.4)	31 (9.6)	17 (5.3)	11,000–15,000 Saudi Riyals
	6 (1.9)	23 (7.1)	11 (3.4)	>15,000 Saudi Riyals
0.000 *	Previous Experience of Breastfeeding with the Last Child			
	4 (2.4)	29 (17.2)	28 (16.6)	Yes
	19 (11.2)	62 (36.7)	27 (16)	No
0.232	Mode of Delivery			
	37 (11.5)	137 (42.5)	67 (20.8)	Vaginal Delivery
	21 (6.5)	36 (11.2)	22 (6.8)	Cesarean Section
0.000 *	Mother’s Plan for Feeding Her Baby			
	4 (1.2)	22 (6.8)	47 (14.6)	Exclusive Breastfeeding
	17 (5.3)	1 (0.3)	1 (0.3)	Formula
	38 (11.8)	150 (46.6)	42 (13)	Mixed
0.240	When Mother Planned How to Feed Her Baby?			
	17 (5.3)	12 (3.7)	6 (1.9)	I did not decide
	18 (5.6)	78 (24.2)	47 (14.6)	Before Pregnancy
	13 (4)	45 (14)	15 (4.7)	During Pregnancy
	8 (2.5)	36 (11.2)	22 (6.8)	After Birth

* Statistically significant (*p* < 0.05).

**Table 2:** Saudi mothers’ knowledge and attitudes about breastfeeding in relation to their infants’ actual feeding practices for the first six months of their infants’ lives in Riyadh, Saudi Arabia.

Mothers’ Knowledge and Attitudes		Exclusive Breastfeeding	Mixed Feeding	Formula Feeding	*p*-Value
	Total	90 (28)	173 (53.7)	59 (18.3)	
I feel that I can overcome breastfeeding problems.	Agree	66 (20.5)	112 (34.8)	31 (9.6)	0.909
	Neither	11 (3.4)	34 (10.6)	13 (4)	
	Disagree	13 (4)	27 (8.4)	15 (4.7)	
I am aware that exclusive breastfeeding is to feed a baby with breast milk without any addition during the first 6 months of the infant’s life.	Agree	69 (21.4)	128 (39.8)	39 (12.1)	0.689
	Neither	6 (1.9)	17 (5.3)	8 (2.5)	
	Disagree	15 (4.7)	28 (8.7)	12 (3.7)	
Breastfeeding helps me to get rid of additional weight that has been gained during the pregnancy period.	Agree	70 (21.7)	131 (40.7)	44 (13.7)	0.387
	Neither	13 (4)	26 (8.1)	7 (2.2)	
	Disagree	7 (2.2)	16 (5)	8 (2.5)	
Breastfeeding helps the uterus return to its normal position faster.	Agree	79 (24.5)	159 (49.4)	51 (15.8)	0.785
	Neither	9 (2.8)	12 (3.7)	6 (1.9)	
	Disagree	2 (0.6)	2 (0.6)	2 (0.6)	
Breastfeeding helps to prevent breast and ovarian cancer.	Agree	75 (23.3)	147 (45.7)	52 (16.1)	0.902
	Neither	12 (3.7)	24 (7.5)	6 (1.9)	
	Disagree	3 (0.9)	2 (0.6)	1 (0.3)	
Breastfeeding helps to prevent getting pregnant.	Agree	54 (16.8)	97 (30.1)	37 (11.5)	0.4880
	Neither	22 (6.8)	46 (14.3)	11 (3.4)	
	Disagree	14 (4.3)	30 (9.3)	11 (3.4)	
Breastfeeding helps to enhance the relationship and increase familiarity with my child.	Agree	86 (26.7)	162 (50.3)	52 (16.1)	0.472
	Neither	3 (0.9)	9 (2.8)	5 (1.6)	
	Disagree	1 (0.3)	2 (0.6)	2 (0.6)	
Breastfeeding helps to improve my body image.	Agree	44 (13.7)	105 (32.6)	33 (10.2)	0.069
	Neither	33 (10.2)	45 (14)	14 (4.3)	
	Disagree	13 (4)	23 (7.1)	12 (3.7)	
Breastfeeding helps to improve general health.	Agree	74 (23)	132 (41)	45 (14)	0.358
	Neither	11 (3.4)	29 (9)	11 (3.4)	
	Disagree	5 (1.6)	12 (3.7)	3 (0.9)	
I think I might stop exclusive breastfeeding if the milk is insufficient.	Agree	28 (8.7)	109 (33.9)	30 (9.3)	0.011 *
	Neither	12 (3.7)	17 (5.3)	8 (2.5)	
	Disagree	50 (15.5)	47 (14.6)	21 (6.5)	
Problems with the nipple or breast may force me to stop breastfeeding.	Agree	25 (7.8)	73 (22.7)	29 (9)	0.089
	Neither	13 (4)	14 (4.3)	7 (2.2)	
	Disagree	52 (16.1)	86 (26.7)	23 (7.1)	
I think that breastfeeding may distort my breast shape.	Agree	16 (5)	33 (10.2)	19 (5.9)	0.423
	Neither	16 (5)	22 (6.8)	9 (2.8)	
	Disagree	58 (18)	118 (36.6)	31 (9.6)	
When I get sick, I stop breastfeeding my infant.	Agree	22 (6.8)	76 (23.6)	28 (8.7)	0.000 *
	Neither	4 (1.2)	21 (12.1)	11 (18.6)	
	Disagree	64 (19.9)	76 (23.6)	20 (6.2)	
When my infant gets sick, I stop breastfeeding my infant.	Agree	17 (5.3)	30 (9.3)	13 (4)	0.071
	Neither	5 (1.6)	8 (2.5)	10 (3.1)	
	Disagree	68 (21.1)	135 (41.9)	36 (11.2)	
I think that breastfeeding is more clean, easy, and available.	Agree	81 (25.2)	160 (49.7)	53 (16.5)	0.905
	Neither	3 (0.9)	2 (0.6)	2 (0.6)	
	Disagree	6 (1.9)	11 (3.4)	4 (1.2)	
I think that breastfeeding is more economical and less expensive than formula milk.	Agree	71 (22)	150 (46.6)	47 (14.6)	0.443
	Neither	8 (2.5)	6 (3.5)	4 (1.2)	
	Disagree	11 (3.4)	17 (5.3)	8 (2.5)	
Breastfeeding infants are less prone to getting diarrhea than an infant who receives formula milk.	Agree	76 (23.6)	152 (47.2)	47 (14.6)	0.410
	Neither	8 (2.5)	16 (5)	8 (2.5)	
	Disagree	6 (1.9)	5 (1.6)	4 (1.2)	
Breastfeeding infants are less prone to getting ear infections than an infant who receives formula milk.	Agree	78 (24.2)	152 (47.2)	50 (15.5)	0.893
	Neither	9 (2.8)	14 (4.3)	7 (2.2)	
	Disagree	3 (0.9)	7 (2.2)	2 (0.6)	
Breastfeeding infants are less prone to getting chest infections than an infant who receives formula milk.	Agree	74 (23)	134 (41.6)	43 (13.4)	0.323
	Neither	11 (3.4)	29 (9)	12 (3.7)	
	Disagree	5 (1.6)	10 (3.1)	4 (1.2)	
Breastfeeding infants are less prone to getting constipation than an infant who receives formula milk.	Agree	75 (23.3)	150 (46.6)	49 (15.2)	0.928
	Neither	10 (3.1)	15 (4.7)	7 (2.2)	
	Disagree	5 (1.6)	8 (2.5)	3 (0.9)	
Breastfeeding infants are less prone to getting colds than infants who receive formula milk.	Agree	66 (20.5)	125 (38.8)	41 (12.7)	0.053
	Neither	19 (5.9)	30 (9.3)	8 (2.5)	
	Disagree	5 (1.6)	18 (5.6)	10 (3.1)	
Breastfeeding infants are less prone to getting food allergies than an infant who receives formula milk.	Agree	66 (20.5)	129 (40.1)	37 (11.5)	0.830
	Neither	17 (5.3)	35 (10.9)	13 (4)	
	Disagree	7 (2.2)	9 (2.8)	9 (2.8)	
Breastfeeding infants are less prone to getting asthma than infants who receive formula milk.	Agree	63 (19.6)	113 (35.1)	36 (11.2)	0.059
	Neither	21 (6.5)	39 (12.1)	13 (4)	
	Disagree	6 (1.9)	21 (6.5)	10 (3.1)	
Breastfeeding infants are smarter than an infant who receives formula feed.	Agree	60 (18.6)	119 (37)	35 (10.9)	0.810
	Neither	21 (6.5)	43 (13.4)	16 (5)	
	Disagree	9 (2.8)	11 (3.4)	8 (2.5)	
Breastfeeding infants are quieter than an infant who receives formula feed.	Agree	54 (16.8)	98 (30.4)	32 (9.9)	0.687
	Neither	24 (7.5)	55 (17.1)	18 (5.6)	
	Disagree	12 (3.7)	20 (6.2)	9 (2.8)	
Breastfeeding infants are healthier than an infant who receives formula feed.	Agree	78 (24.2)	147 (45.7)	52 (16.1)	0.255
	Neither	10 (3.1)	18 (5.6)	4 (6.8)	
	Disagree	2 (0.6)	8 (2.5)	3 (0.9)	
Breastfeeding infants are less obese than an infant who receive formula feed.	Agree	67 (20.8)	121 (37.6)	35 (10.9)	0.176
	Neither	15 (4.7)	35 (10.9)	18 (5.6)	
	Disagree	8 (2.5)	17 (5.3)	6 (1.9)	

* Statistically significant: *p* < 0.05; data are given as a percentage.

**Table 3:** Associations of Saudi mothers’ degree of comfort in breastfeeding in social situations with their infants’ actual feeding practices for the first six months of their infants’ lives in Riyadh, Saudi Arabia.

Mothers’ Degree of Comfort When Breastfeeding in Social Situations		Exclusive Breastfeeding	Mixed Feeding	Formula Feeding	*p*-Value
	Total	90 (28)	173 (53.7)	59 (18.3)	
I can breastfeed my infant in front of my relative.	Agree	51 (15.8)	82 (25.5)	18 (5.6)	0.024 *
	Neither	8 (2.5)	14 (4.3)	7 (2.2)	
	Disagree	31 (9.6)	77 (23.9)	34 (10.6)	
I can breastfeed in front of my best friends.	Agree	49 (15.2)	78 (24.2)	19 (5.9)	0.028 *
	Neither	12 (3.7)	15 (4.7)	9 (2.8)	
	Disagree	29 (9)	80 (24.8)	31 (9.6)	
I can breastfeed in public.	Agree	20 (6.2)	23 (7.1)	10 (3.1)	0.132
	Neither	9 (2.8)	12 (3.7)	4 (1.2)	
	Disagree	61 (18.9)	138 (42.9)	45 (14)	
Our society promotes and supports breastfeeding practice.	Agree	59 (18.3)	122 (37.9)	42 (13)	0.806
	Neither	14 (4.3)	25 (7.8)	8 (2.5)	
	Disagree	17 (5.3)	26 (8.1)	9 (2.8)	
The absence of a suitable place for breastfeeding caused me to stop breastfeeding.	Agree	36 (11.2)	98 (30.4)	25 (7.8)	0.043 *
	Neither	10 (3.1)	28 (8.7)	11 (3.4)	
	Disagree	44 (13.7)	47 (14.6)	23 (7.1)	

* Statistically significant: *p* < 0.05; data are given as a percentage.

**Table 4:** Multinomial logistic regression analysis of factors associated with exclusive breastfeeding at the age of 6 months in Riyadh.

Feeding Type/Variable		B	SE	Wald	df	Sig	OR	95% CI	
Exclusive Breastfeeding	Antenatal infant’s feeding plan								
	Exclusive breastfeeding	1.99	0.603	10.9	1	0.001	7.309	2.241	23.839
	Exclusive formula feeding	−3.192	1.118	8.156	1	0.004	0.041	0.005	0.367
	Mixed feeding	REF							
	Previous breastfeeding experience								
	None	−1.226	0.643	3.631	1	0.057	0.294	0.083	1.036
	Yes	REF							
	Ability to breastfeed in front of relatives								
	Yes	−1.257	0.946	1.764	1	0.184	0.285	0.045	1.818
	No	−1.649	0.968	2.900	1	0.089	0.192	0.029	1.283
	Not sure	REF							
	Ability to breastfeed in front of best friends								
	Yes	0.864	0.826	1.094	1	0.296	2.373	0.470	11.984
	No	0.619	0.846	0.536	1	0.464	1.857	0.354	9.747
	Not sure	REF							
	The absence of suitable places for breastfeeding made me stop breastfeeding								
	Yes	0.508	0.671	0.573	1	0.449	1.662	0.446	6.197
	No	0.669	0.692	0.933	1	0.334	1.952	0.503	7.576
	Not sure	REF							
	Stop breastfeeding if milk insufficient								
	Yes	−0.646	0.666	0.941	1	0.332	0.524	0.142	1.934
	No	−0.042	0.685	0.004	1	0.951	0.959	0.250	3.671
	Not sure	REF							
	Stop breastfeeding if mother gets sick								
	Yes	0.788	0.823	0.915	1	0.339	2.198	0.438	11.042
	No	1.620	0.808	4.020	1	0.045	5.054	1.037	24.627
	Not sure	REF							
Mixed feeding	Antenatal infant’s feeding plan								
	Exclusive breastfeeding	0.172	0.597	0.083	1	0.773	1.188	0.369	3.827
	Exclusive formula feeding	−4.305	1.079	15.913	1	0.000	0.013	0.002	0.112
	Mixed feeding	REF							
	Previous breastfeeding experience								
	None	−0.817	0.609	1.799	1	0.180	0.442	0.134	1.458
	Yes	REF							
	Ability to breastfeed in front of relatives								
	Yes	−0.365	0.856	0.182	1	0.670	0.694	0.130	3.718
	No	−1.032	0.861	1.434	1	0.231	0.356	0.066	1.928
	Not sure	REF							
	Ability to breastfeed in front of best friends								
	Yes	1.155	0.733	2.479	1	0.115	3.173	0.754	13.362
	No	1.041	0.732	2.024	1	0.155	2.832	0.675	11.885
	Not sure	REF							
	The absence of suitable places for breastfeeding made me stop breastfeeding								
	Yes	0.124	0.528	0.055	1	0.814	1.132	0.402	3.183
	No	−0.401	0.557	0.519	1	0.471	0.670	0.225	1.994
	Not sure	REF							
	Stop breastfeeding if milk insufficient								
	Yes	0.558	0.585	0.910	1	0.340	1.747	0.555	5.494
	No	0.143	0.626	0.052	1	0.819	1.154	0.338	3.932
	Not sure	REF							
	Stop breastfeeding if mother gets sick								
	Yes	−0.436	0.581	0.563	1	0.453	0.646	0.207	2.020
	No	0.035	0.596	0.003	1	0.954	1.035	0.322	3.326
	Not sure	REF							

## Data Availability

The datasets generated and analyzed during the current study are available from the corresponding author on reasonable request.
